# Supplementary material for: The causal role of circulating inflammatory markers in osteoporosis: a bidirectional Mendelian randomized study
Source: Front Immunol. 2024 Jul 18;15:1412298. doi: 10.3389/fimmu.2024.1412298 (PMC11291241; doi:10.3389/fimmu.2024.1412298)
Supplement: Supplementary file 1 [file DataSheet_1.zip › supplementary material/CIM-OSTEOPOROSIS /GCST90274823.csv_forest.pdf]

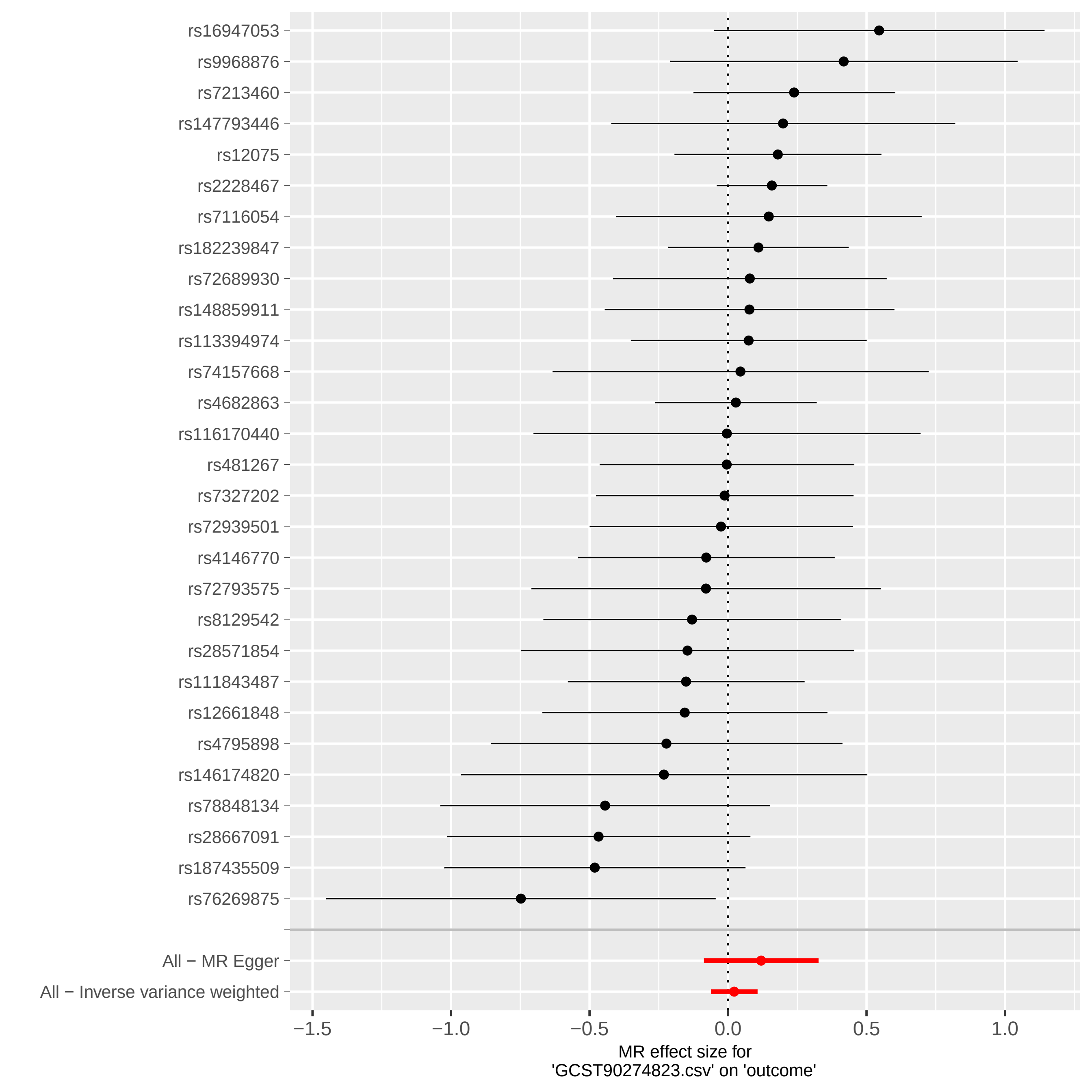
rs16947053
rs9968876
rs7213460
rs147793446
rs12075
rs2228467
rs7116054
rs182239847
rs72689930
rs148859911
rs113394974
rs74157668
rs4682863
rs116170440
rs481267
rs7327202
rs72939501
rs4146770
rs72793575
rs8129542
rs28571854
rs111843487
rs12661848
rs4795898
rs146174820
rs78848134
rs28667091
rs187435509
rs76269875
All − MR Egger
All − Inverse variance weighted
−1.5
−1.0
−0.5
0.0
0.5
1.0
MR effect size for
'GCST90274823.csv' on 'outcome'
